# Alcohol consumption habits and associations with anxiety or depressive symptoms postpartum in women with high socioeconomic status in Sweden

**DOI:** 10.1007/s00737-022-01268-y

**Published:** 2022-09-26

**Authors:** Susanne Lager, Karin Gidén, Cathrine Axfors, Frida Sigvardsson, Natasa Kollia, Ingrid Nylander, Emma Fransson, Alkistis Skalkidou

**Affiliations:** 1 Department for Women’s and Children’s Health, Uppsala University, Uppsala, Sweden; 2 Department of Microbiology, Tumor and Cell Biology, Centre for Translational Microbiome Research, Karolinska Institutet, Stockholm, Sweden; 3 Meta-Research Innovation Center at Stanford (METRICS), Stanford, CA USA; 4 Department of Pharmaceutical Biosciences, Uppsala University, Uppsala, Sweden; 5 Department of Nutrition and Dietetics, School of Health Science and Education, Harokopio University, Athens, Greece

**Keywords:** Alcohol drinking, Depression, Postpartum, Anxiety, Pregnancy, Mothers

## Abstract

Postpar tum depression and anxiety are common among new mothers. It is well-established that in the general population alcohol use is associated with depression and anxiety. Linking alcohol consumption to symptoms of postpartum depression (PPDS) or postpartum anxiety (PPAS) is presently less established. This study aims to determine if alcohol consumption pre-pregnancy, 6 weeks postpartum, 6 months postpartum, or changes in alcohol consumption are associated with PPDS or PPAS. Longitudinal data on 3849 women from a Swedish perinatal cohort were analyzed using logistic regression analyses for associations between alcohol consumption and symptoms of anxiety or depression, as assessed with the Edinburgh Postnatal Depression Scale. There was no association between pre-pregnancy drinking habits and PPDS (*p* = 0.588, *n* = 2479) or PPAS (*p* = 0.942; *n* = 2449) at 6 weeks postpartum. Similarly, no associations were observed between concurrent drinking habits at 6 weeks postpartum and PPAS (*p* = 0.070, *n* = 3626), 6 months postpartum and PPDS (0.647, *n* = 3461) or PPAS (*p* = 0.700, *n* = 3431). However, there was an association between drinking habits at 6 weeks postpartum and concurrent PPDS (*p* = 0.047, *n* = 3659). In conclusion, robust associations were not found between postpartum alcohol consumption and mood symptoms. This lack of association between poor mental health and risk behaviors in new mothers could be interpreted as a result of long-term policy work and high participation in Swedish maternity care. Future studies need to address these research questions in more diverse socio-cultural contexts.

## Introduction

Postpartum depression (PPD) is a depressive episode, with typical onset within 4 weeks after childbirth. This is a serious mental health problem affecting almost one in eight of all new mothers (O'Hara and McCabe, 2013). PPD expresses itself across a range of symptoms: depressed mood, disturbances of appetite and sleep, feelings of inadequacy, as well as decreased interest in social activities. Severe cases of PPD may result in self-destructive behaviors, harm/neglect of the child, and possibly suicide. Along with symptoms of PPD (PPDS), anxiety is common for new mothers. It is estimated between 17 and 30% of women experience symptoms of anxiety postpartum (PPAS) (Fairbrother et al. 2016; Wenzel et al. 2005). There is notable comorbidity between PPAS and PPDS (Miller et al. 2015; Wenzel et al. 2005). In the early postpartum period, such comorbidity affects about 5–13% of women (Fairbrother et al. 2016; Falah-Hassani et al. 2016). A bi-directional relationship between anxiety and depression exists insomuch as presence of one increases the risk for the other (K. A. Grant et al. 2008; Mauri et al. 2010; Skouteris et al. 2009).

The link between depression or anxiety, with alcohol use, outside the peripartum period is well-established. Both anxiety and depression often occur together with alcohol use disorder (Boden and Fergusson, 2011; B. F. Grant et al. 2015; McHugh and Weiss, 2019). In a longitudinal study of young people, reduction in alcohol use with age paralleled a decreasing prevalence of mental disorders (Gustavson et al. 2018). The World Health Organization (WHO) reports a mean yearly alcohol consumption of 6.4 L pure alcohol per person. However, drinking habits differ worldwide (WHO, 2018). In Sweden, the mean yearly alcohol consumption is 9.2 L pure alcohol per person and 80% of the adult population reported consuming alcohol the previous month (Guttormsson, 2020; WHO, 2018). Regional differences in alcohol consumption during the peripartum period also exist. A study of approximately 7900 women in eleven European countries stated that alcohol use when pregnant was most common among UK women and least common among Norwegian women (Mardby et al. 2017). This indicates that traditional and cultural differences in alcohol consumption may affect peripartum alcohol use. Furthermore, such differences may also impact the drinking habits of women experiencing depressive symptoms and anxiety.

At present, very few published articles explore associations between alcohol consumption and postpartum anxiety. A descriptive study reported higher alcohol consumption in women with PPAS, compared to PPDS or comorbid conditions (Farr et al. 2014). Studies examining the relationship between alcohol consumption and PPD show mixed results. Some studies have associated alcohol use (from any alcohol use to problematic drinking habits, such as alcohol use disorder) with a higher risk of PPD (Badr et al. 2018; Peltzer et al. 2018; Theme Filha et al. 2016). In contrast, women suffering from PPD have also been reported being less likely to drink alcohol during pregnancy (Katon et al. 2014). Another study found alcohol use pre-pregnancy was associated with a higher risk of PPD in adolescent women, while adult women using alcohol had a lower risk of PPD than those who abstained (Nunes and Phipps, 2013). Of importance is the fact that several studies investigating associations between alcohol use and PPDS or PPAS have focused on risk groups. Therefore, for the general population, it is less known if, and how, alcohol consumption before and through the perinatal period and PPDS or PPAS are associated. Study aims were to establish if PPAS/PPDS are associated with alcohol consumption before pregnancy and at 6 weeks/6 months postpartum and with changes in consumption during these time periods.

## Methods

### Participants

Women receiving ultrasound dating scans at Uppsala University Hospital, Sweden, were eligible to participate in the population-based *Biology, Affect, Stress, Imaging and Cognition* (BASIC) cohort study. A detailed description of the BASIC cohort has been published (Axfors et al. 2019). Briefly, the study included several online surveys during pregnancy and postpartum, along with collection of biological samples and information from perinatal medical records.

**Fig. 1:**
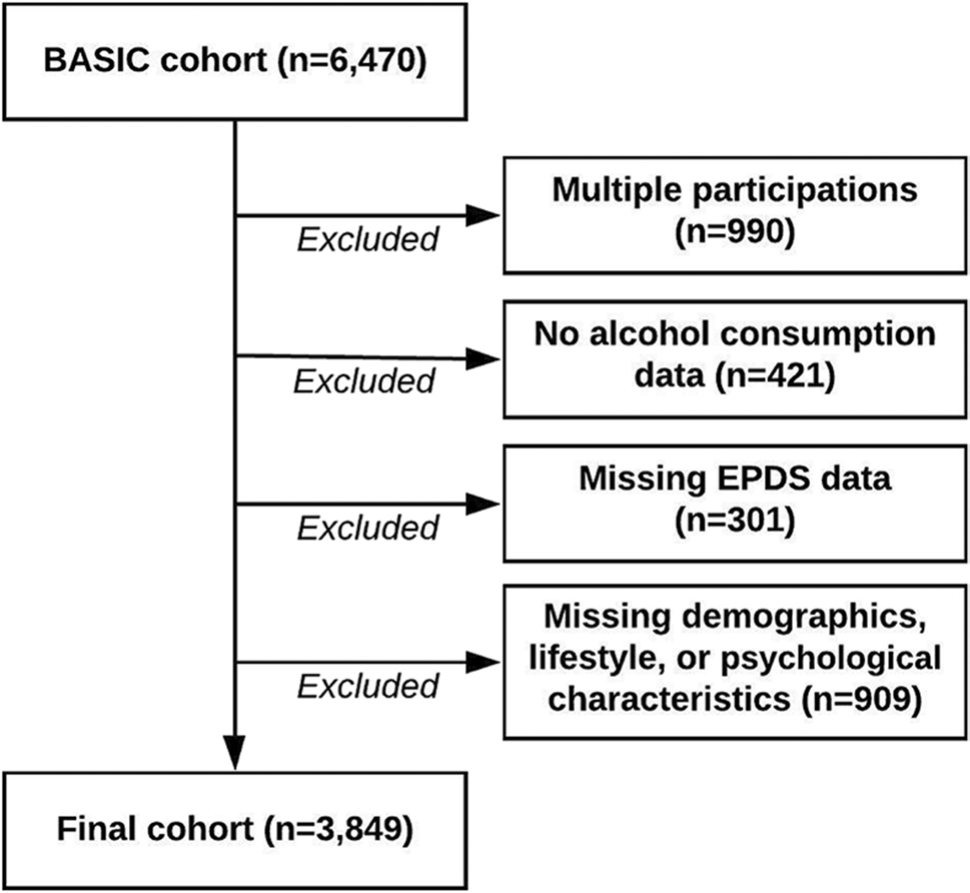
Flow chart of selection of study participants. From the BASIC cohort, data on 6470 pregnancies were available. For women participating more than once in the BASIC cohort, only data from their first participation was included in the current study analysis. All participants without any alcohol consumption data at either before pregnancy, at 6 weeks postpartum, or 6 months postpartum were excluded. Further, all participants missing EPDS data at 6 weeks postpartum (for analyses of pre-pregnancy and 6 weeks postpartum drinking habits) or 6 months postpartum (for analyses with 6 months postpartum drinking habits) were omitted from the analysis. All participants missing breastfeeding or partner support data at 6 weeks postpartum (for analyses of pre-pregnancy and 6 weeks postpartum drinking habits) or 6 months postpartum (for analyses with 6 months postpartum drinking habits) were omitted from the analysis. Lastly, all participants with missing information on age, education level, birth region, parity previous alcohol dependency, or history of depression were excluded. The final cohort consisted of 3849 participants

At enrollment in the BASIC cohort, participants answered questions about demographic, lifestyle, and psychological characteristics. Information regarding drinking habits at 3 months pre-pregnancy was obtained from medical records, originally collected by midwives at the first visit for routine antenatal care. Based on nationwide Swedish data, this initial visit usually took place around pregnancy week 9–10 during the years of cohort recruitment (Swedish Pregnancy Register, 2020). At 6 weeks and 6 months postpartum, participants reported current alcohol consumption (open-ended question), how infants were fed (breastfeeding exclusively, bottle exclusively, or a combination), perceived support by partner in caring for the infant, and answered the Edinburgh Postnatal Depression Scale (EPDS) questionnaire (Cox et al. 1987). For women participating in the BASIC cohort more than once, only data from their first participation was included in this study. Women were excluded if data was missing concerning alcohol consumption, EPDS score, education level, maternal age, maternal birth region, parity, previous alcohol dependency, and history of depression. The final cohort consisted of 3849 participants (Fig. 1).

### Exposures

Frequency of alcohol consumption (at 3 months before pregnancy, 6 weeks postpartum, and 6 months postpartum) was classified into three groups: “Never/Seldom,” “Once a week,” and “More than once a week.” Changes in frequency of alcohol consumption (from pregnancy to 6 weeks postpartum and from 6 weeks postpartum to 6 months postpartum) were grouped into three categories (based on the frequency of alcohol consumption groups): “no change,” “decreased drinking,” and “increased drinking.” In the “no change” category, women consumed alcohol at a similar frequency for both time points. In the “decreased drinking” category, women consumed less frequently at the later time point compared to the earlier time point. For the “increased drinking” category, women consumed alcohol more often at the later time point compared to the earlier time point.

### Outcomes

The EPDS questionnaire was used to define PPDS and PPAS (Cox et al. 1987). Specifically, significant PPDS were defined as a score of 12 or above on a total of 10 questions (Levis et al. 2020), while significant PPAS was an EPDS-A-subscale score ≥ 6 (subscale including items 3–5) (Matthey et al. 2013).

### Statistical analysis

Descriptive statistics are presented as numbers and percentages. Shapiro–Wilk’s test was used to assess normality of data distribution in EPDS scores. The Kruskal–Wallis test was used to assess univariable associations of EPDS scores and the three alcohol consumption categories. To assess associations between alcohol consumption and PPDS or PPAS, binary logistic regressions were used (drinking once a week was used as reference category). Models were adjusted for breastfeeding, education level, maternal age, maternal birth region, parity, partner support, previous alcohol dependency, and history of depression. Additional models were run assessing associations between alcohol consumption and PPDS or PPAS at 6 months postpartum and adjusted for the above-mentioned factors, along with concurrent selective serotonin reuptake inhibitors (SSRI) treatment. As a sensitivity analysis, analyses were repeated while excluding the ten women with previous alcohol dependency. Associations were expressed as an odds ratio (OR) with 95% confidence intervals (CIs). *p* values below 0.05 were considered statistically significant. All analyses were performed with IBM SPSS Statistics (version 27.0).

## Results

### Demographic, lifestyle, and psychological characteristics

Most women in this study were born in Scandinavia, had university level education, and were married or co-habiting with their partner. Almost a third self-reported a history of depression (*n* = 3849; Table 1).

**Table 1:** Demographic, lifestyle, and psychological characteristics of study cohort (*n* = 3849)

Variable	*n* (%)
Maternal age	
< *25 years*	231 (6.0%)
* 25–34 years*	2715 (70.5%)
≥ *35 years*	903 (23.5%)
Maternal birth region	
* Scandinavia*	3561 (92.5%)
* Other*	288 (7.5%)
Education level	
* University*	2973 (77.2%)
* No university*	876 (22.8%)
Working in early pregnancy	
* Full time*	2462 (64.0%)
* Part time*	763 (19.8%)
* Student*	272 (7.1%)
* Parental leave*	124 (3.2%)
* Sick leave*	105 (2.7%)
* Unemployed*	114 (3.0%)
* Missing*	9 (0.2%)
Marital status at 6 weeks postpartum	
* Married/cohabiting*	3689 (95.8%)
* Single*	46 (1.2%)
* Missing*	114 (3.0%)
Parity	
* Primipara*	2190 (56.9%)
* Multipara*	1659 (43.1%)
Body mass index (kg/m^2^)	
< *25*	2766 (71.9%)
* 25–30*	751 (19.5%)
> *30*	320 (8.3%)
* Missing*	12 (0.3%)
History of depression	
* No*	2699 (70.1%)
* Yes*	1150 (29.9%)
Previous dependency of alcohol	
* No*	3839 (99.7%)
* Yes*	10 (0.3%)
Drinking habits 3 months before pregnancy	
* Never/seldom*	1497 (38.9%)
* Once a week*	863 (22.4%)
* More than once a week*	227 (5.9%)
* Missing*	1262 (32.8%)
Drinking habits 6 weeks postpartum	
* Never/seldom*	2887 (75.0%)
* Once a week*	587 (15.3%)
* More than once a week*	219 (5.7%)
* Missing*	156 (4.1%)
Drinking habits 6 months postpartum	
* Never/seldom*	2479 (64.4%)
* Once a week*	769 (20.0%)
* More than once a week*	264 (6.9%)
* Missing*	337 (8.8%)
Smoking ever	
* No*	2591 (67.3%)
* Yes*	1231 (32.0%)
* Missing*	27 (0.7%)
Breastfeeding at 6 weeks postpartum	
* Yes full time*	2845 (73.9%)
* Yes and also bottle feed*	622 (16.2%)
* No*	268 (7.0%)
* Missing*	114 (3.0%)
Breastfeeding at 6 months postpartum	
* Yes full time*	1016 (26.4%)
* Yes and also bottle feed*	1693 (44.0%)
* No*	841 (21.8%)
* Missing*	299 (7.8%)
Partner support at 6 weeks postpartum	
* Yes much help*	2336 (60.7%)
* Yes some help*	1280 (33.2%)
* No*	112 (2.9%)
* Missing*	121 (3.1%)
Partner support at 6 months postpartum	
* Yes much help*	2147 (55.8%)
* Yes some help*	1265 (32.9%)
* No*	130 (3.4%)
* Missing*	307 (8.0%)

### Frequency of alcohol consumption

Among women using alcohol, most reported drinking once a week. A smaller proportion reported consumption of alcohol several times a week (Table 1). Alcohol consumption data prior to pregnancy was available for 2587 women. Among these women, 42.2% reported that they consumed alcohol once a week or more. Postpartum, the proportion of women consuming alcohol frequently was lower. At 6 weeks postpartum, alcohol consumption data was available for 3693 women, with 21.8% of these women reporting consumption of alcohol once a week or more. At 6 months postpartum, 29.4% of the 3512 women reported alcohol consumption of once a week or more.

### Alcohol consumption and depressive symptoms

**Fig. 2:**
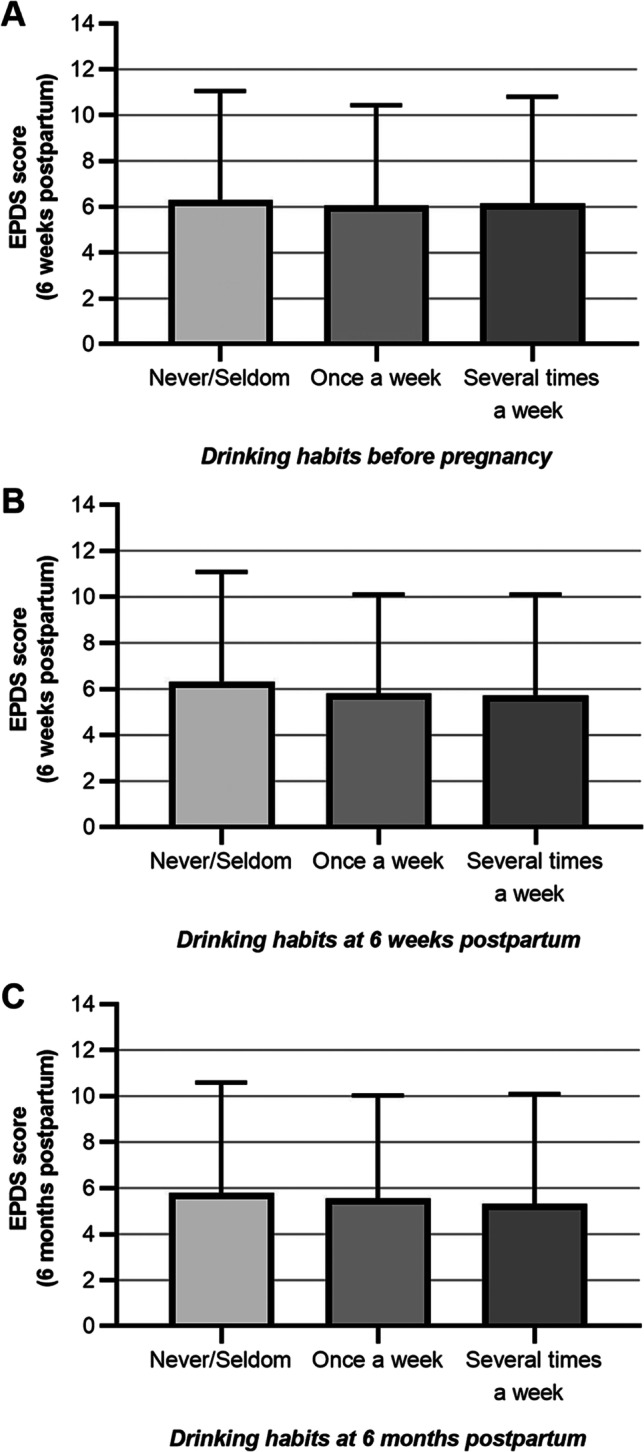
Drinking habits before pregnancy (**A**), at 6 weeks postpartum (**B**), and at 6 months postpartum (**C**) and EPDS scores postpartum. Differences between groups were assessed with the Kruskal–Wallis test (all results *p* > 0.05). Bars represent mean values and error bars represent standard deviations

**Fig. 3:**
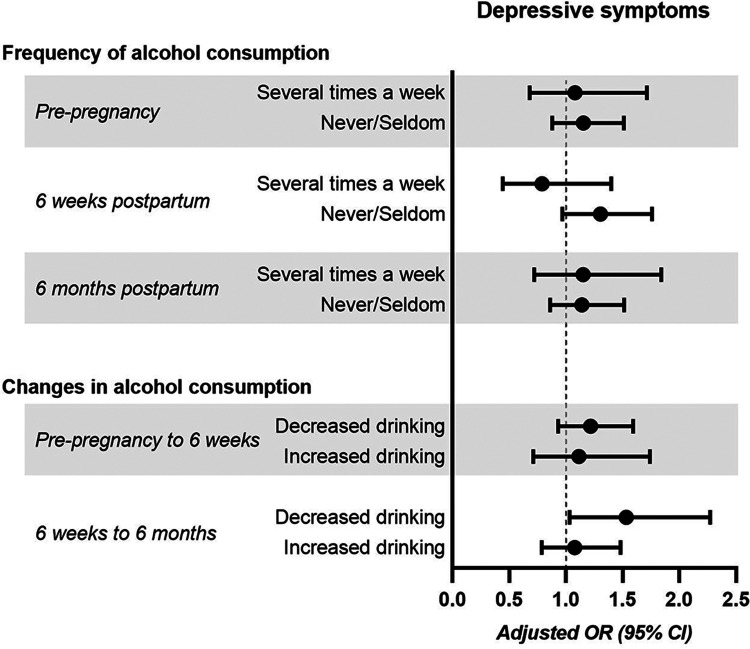
Depressive symptoms and drinking habits. Frequency or change in alcohol consumption and odds ratio for depressive symptoms postpartum (EPDS ≥ 12). Drinking alcohol once a week was used as reference in the upper panel, and no change in drinking habits was the reference in the lower panel. Depressive symptoms were assessed at 6 weeks postpartum (for pre-pregnancy alcohol consumption, concurrent alcohol consumption, and changes in alcohol consumption from pre-pregnancy to 6 weeks postpartum), or at 6 months postpartum (for concurrent frequency of alcohol consumption and changes in alcohol consumption from 6 weeks to 6 months postpartum). Logistic regression models were adjusted for breastfeeding, education level, maternal age, maternal birth region, parity, partner support, previous alcohol dependency, and history of depression

Approximately 10% of women showed signs of depression, according to the EPDS scores (Table 2). There were no univariable associations between PPDS and the three groups based on alcohol consumption habits (Kruskal–Wallis test, *p* > 0.05; Fig. 2). In the adjusted logistic regression analysis, there was no association between pre-pregnancy drinking habits and PPDS at 6 weeks postpartum (*p* = 0.588; *n* = 2479). Overall, when comparing all three groups, there was an association between drinking habits and concurrent PPDS 6 weeks postpartum (*p* = 0.047; *n* = 3659). Compared to women consuming alcohol once a week, women never/seldom consuming alcohol at 6 weeks postpartum had an adjusted OR of 1.3 for PPDS, but this pairwise sub-comparison was not statistically significant (Fig. 3). At 6 months postpartum, there was no association between alcohol consumption and PPDS (*p* = 0.647; *n* = 3461). Excluding women with previous alcohol dependency had no major impact on findings (data not shown), except that the association between drinking habits and PPDS at 6 weeks postpartum was no longer statistically significant (*p* = 0.053; *n* = 3651).

**Table 2:** Symptoms of anxiety and depression in study cohort (*n* = 3849)

Variable	*n* (%)
Anxiety at 6 weeks postpartum (EPDS-A-subscale score ≥ 6)	
* No*	3384 (87.9%)
* Yes*	290 (7.5%)
* Missing*	175 (4.5%)
Anxiety at 6 months postpartum (EPDS-A-subscale score ≥ 6)	
* No*	3247 (84.4%)
* Yes*	227 (5.9%)
* Missing*	375 (9.7%)
Depressive symptoms at pregnancy week 17 (EPDS score ≥ 13)	
* No*	3482 (90.5%)
* Yes*	354 (9.2%)
* Missing*	13 (0.3%)
Depressive symptoms at pregnancy week 32 (EPDS score ≥ 13)	
* No*	3358 (87.2%)
* Yes*	354 (9.2%)
* Missing*	137 (3.6%)
Depressive symptoms at 6 weeks postpartum (EPDS score ≥ 12)	
* No*	3224 (83.8%)
* Yes*	484 (12.6%)
* Missing*	141 (3.7%)
Depressive symptoms at 6 months postpartum (EPDS score ≥ 12)	
* No*	3094 (80.4%)
* Yes*	411 (10.7%)
* Missing*	344 (8.9%)
SSRI treatment at 6 months postpartum	
* No*	3238 (84.1%)
* Yes*	187 (4.9%)
* Missing*	424 (11.0%)

*EPDS*, Edinburgh Postnatal Depression Scale; *SSRI*, selective serotonin reuptake inhibitors

### Changes in frequency of alcohol consumption and depressive symptoms

A total of 2482 women reported their drinking habits before pregnancy and at 6 weeks postpartum. Of these women, most reported no change in drinking frequency between the two time points. However, 8.8% reported they consumed alcohol more often at 6 weeks postpartum compared to pre-pregnancy, while 28.9% consumed alcohol less frequently. There was no association between change in drinking habits and PPDS at 6 weeks postpartum (*p* = 0.351; *n* = 2455; Fig. 3).

A total of 3362 women reported drinking habits at both 6 weeks and 6 months postpartum. Most of these women did not change alcohol consumption habits between the two time points. A minority did report changed habits (14.9% consuming alcohol more, 7.3% consuming less) at 6 months postpartum compared to at 6 weeks postpartum. Overall, there was no association between a change in drinking habits and PPDS at 6 months postpartum, when comparing all three groups (*p* = 0.103; *n* = 3312). However, when only comparing women of decreased frequency of alcohol consumption with those of unchanged drinking habits, the women that drank less were at an increased risk for PPDS (adjusted OR 1.5 (95% CI 1.01–2.27), *p* = 0.034; Fig. 3). When including SSRI treatment in an additional model, the association between decreased frequency of drinking with increased risk of PPDS was attenuated, being no longer statistically significant (adjusted OR 1.4, 95% CI 0.94–2.13; *p* = 0.101; *n* = 3183).

### Alcohol consumption and anxiety

**Fig. 4:**
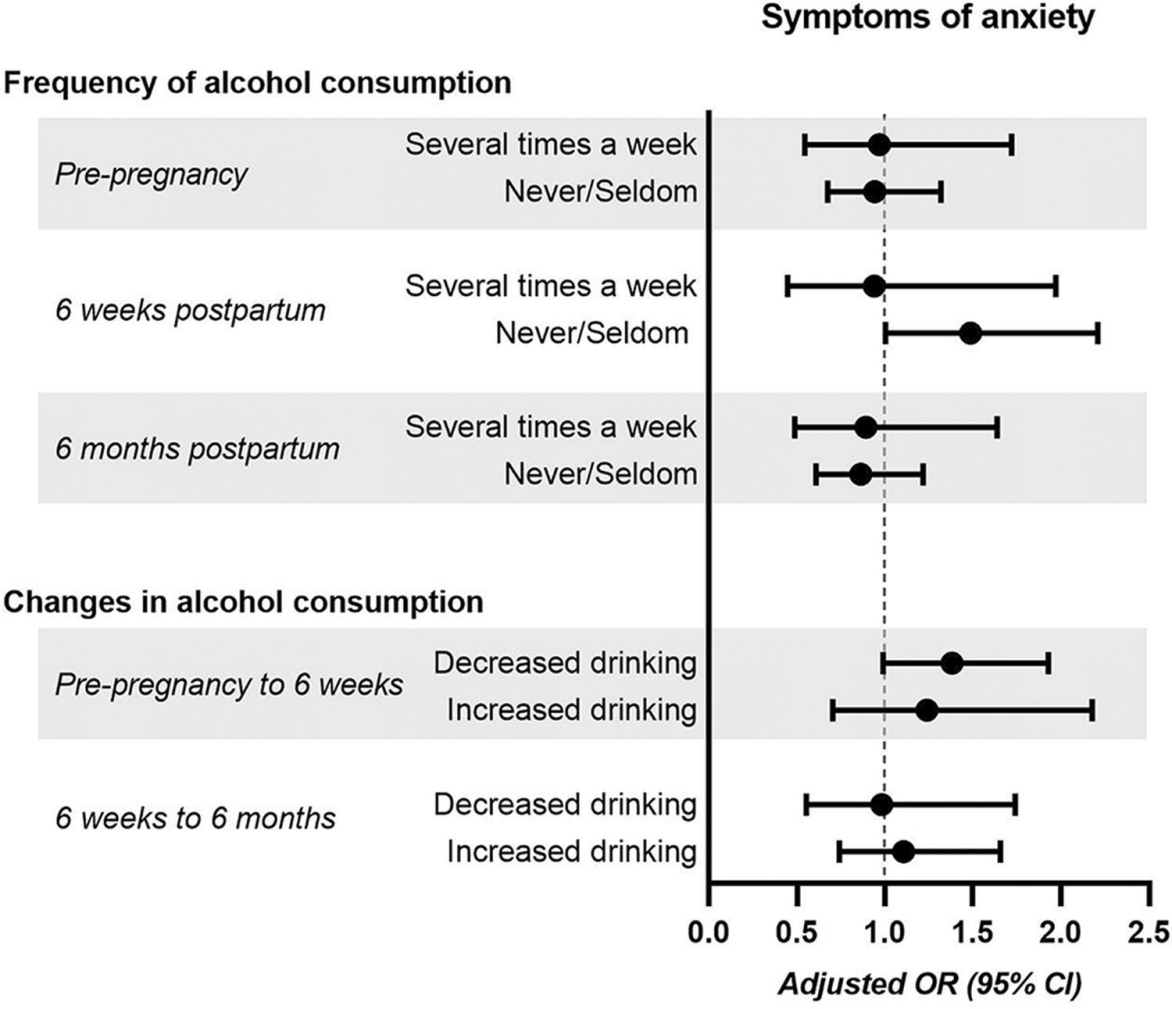
Symptoms of anxiety and drinking habits. Frequency or change in alcohol consumption and odds ratio for symptoms of anxiety postpartum (EPDS-A-subscale score ≥ 6). Drinking alcohol once a week or no change in drinking habits was used as reference group. Symptoms of anxiety were assessed at 6 weeks postpartum (for pre-pregnancy alcohol consumption, concurrent alcohol consumption, and changes in alcohol consumption from pre-pregnancy to 6 weeks postpartum), or at 6 months postpartum (for concurrent frequency of alcohol consumption and changes in alcohol consumption from 6 weeks to 6 months postpartum). Logistic regression models were adjusted for maternal age, maternal birth region, parity, education, history of depression, previous alcohol use disorder, breast-feeding, and partner support

Postpartum, 6–7% of the women showed significant signs of anxiety according to their EPDS-A-subscale scores (Table 2). PPAS at 6 weeks postpartum and frequency of alcohol consumption before pregnancy were not associated (*p* = 0.942; *n* = 2449). Overall, drinking habits at 6 weeks postpartum and concurrent PPAS were not associated (*p* = 0.070; *n* = 3626). When comparing women that never or seldom consumed alcohol to women consuming alcohol once a week, the women in the former group were at increased risk for PPAS (*p* = 0.049; Fig. 4). At 6 months postpartum, there was no association between drinking habits and concurrent PPAS (*p* = 0.700; *n* = 3431). Furthermore, there was no association between PPAS and changes in drinking habits [comparing pre-pregnancy and 6 months postpartum (*p* = 0.154; *n* = 2426), as well as between 6 weeks and 6 months postpartum (*p* = 0.878; *n* = 3282)].

## Discussion

In this study, no robust associations between alcohol consumption and PPDS/PPAS were detected. Two associations were just below the cut-off for statistical significance. Women consuming alcohol less frequently at 6 weeks and 6 months in the postpartum period were at increased risk for PPDS, while those rarely consuming alcohol had an increased risk of PPAS. Because of the many different comparisons in this study, it is possible these are spurious findings. These findings diverge from several previous studies where alcohol consumption or problematic drinking behaviors are linked with an increased risk of PPD (Badr et al. 2018; Peltzer et al. 2018; Theme Filha et al. 2016). The divergent findings could potentially be related to differing cohorts (such as differing educational levels, age, or social backgrounds). There are also important differences in design and methodology; only three of the studies were longitudinal and adjusted for other factors (Badr et al. 2018; Peltzer et al. 2018; Theme Filha et al. 2016). In similarity with our findings, a recent large-scale study of women in Japan observed no association between hazardous drinking habits and postpartum depression (Murakami et al. 2021).

In this study, all measurement points were utilized to explore possible associations. We do not recommend interpreting these as robust associations, but rather as an expected number of statistically significant results in the context of our explorative study. Previous studies have shown that both no drinking and heavy drinking are linked to an increased risk of depression, whereas low or moderate drinking was linked to less-depressive symptoms in non-perinatal cohorts. For instance, one study showed that two to seven glasses of wine per week were associated with lower risk of depression (Gea et al. 2013). Another study indicated that moderate alcohol use is not a risk factor for depression (Paschall et al. 2005). Potentially, decreased social interactions among depressed individuals could be a bidirectional factor (Canham et al. 2016). However, loneliness and associated depression was also linked to an increased prevalence of risky use and binge drinking (Canham et al. 2016). Importantly, these patterns could differ widely between individuals of diverse social and cultural backgrounds (Rehm et al. 2003), as well as within a perinatal setting. Regarding anxiety, we do not believe greater alcohol consumption in postpartum women results in lessening of anxiety/depressive symptoms. A plausible explanation may be that anxiety symptoms are associated with social withdrawal and therefore these women did not participate in social drinking (Canham et al. 2016).

Our study cohort consists of a high proportion of participants (> 77%) with a university education. Individuals from higher socio-economic backgrounds have been reported to be more likely to use alcohol to increase confidence in social situations. In contrast, people from a low socio-economic background were more likely to use alcohol to cope with low mood (Stapinski et al. 2016).

Over 90% of the study participants were born in Scandinavia. Mean alcohol consumption in Scandinavia is higher than worldwide consumption (WHO, 2018). In general, the study cohort could therefore at a higher risk traditionally for greater alcohol consumption when compared to the total world population. This may affect results and therefore data may not be applicable to other countries. Attitudes towards alcohol consumption in the peripartum period appear to differ between ethnic groups. A study comparing women in Sweden found that participants born in the Nordic countries were more likely to consume alcohol before pregnancy and in early pregnancy than women born in or outside Europe (Hultstrand et al. 2020). Yet, Swedish parents have been reported expressing strong views supporting total alcohol abstinence during pregnancy (Scholin et al. 2018). These two studies suggest that our results may not be applicable to countries outside Scandinavia.

Several studies have shown a relationship between depression and substance use disorders, concluding there exists a common co-occurrence of depression and alcohol abuse (Boden and Fergusson, 2011; Swendsen and Merikangas, 2000). Therefore, treatment of one condition could affect the other. With this in mind, exactly how alcohol consumption changes during and after treatment for postpartum depression would be of interest. When adjusted for SSRI use, we see no change in the (non-)association between alcohol consumption and PPDS. The data in the study concerning SSRI use contains no information about the duration of SSRI treatment or when first prescribed, only that it was/was not on the individual’s drug list at that time. Future studies should investigate whether SSRI treatment for women with depressive symptoms in the postpartum period affects alcohol consumption.

### Limitations and strengths

The main limitation of this study is the analysis being based on self-reported frequency of alcohol consumption. Oral interviews or other variants of self-report questions might have uncovered nuances of alcohol consumption habits not available in this study cohort. Previous research has shown self-reporting on alcohol consumption usually means underreporting (Devos-Comby and Lange, 2008). However, other studies have shown self-reporting methods are a reliable approach for measuring alcohol intake (Del Boca and Darkes, 2003). These studies have not been made in perinatal cohorts and may not be fully applicable in our cohort. Another limitation is the absence of measurement of how many units of alcohol were consumed per week and type of alcohol consumed. Data regarding the amount in standard units of alcohol consumed was not collected for the BASIC cohort and therefore such an analysis was not possible.

To the best of our knowledge, this is one of the largest studies so far conducted on this subject in a perinatal sample, drawing data from the longitudinal BASIC cohort. Longitudinal studies have several advantages, such as establishing a sequence of events in specific individuals, registering changes over time, and collecting data prospectively which limits recall bias (Caruana et al. 2015). The BASIC cohort population is homogeneous. The cohort includes few socio-economically disadvantaged women, who are possibly at higher risk for both affective disorder and problematic alcohol use. In addition, dropouts from the BASIC cohort were higher among participants with PPDS compared to those not having PPDS (Axfors et al. 2019).

In the BASIC cohort, self-reporting surveys were used to collect data about depressive symptoms. This is a limitation since self-reported information may include information bias (Devaux and Sassi, 2016). However, the EPDS scoring system used in the psychometric evaluation of participants is widely accepted as a reliable instrument for collecting information about psychological states during the postpartum period (Cox et al. 1987).

## Conclusion

We found no robust associations between alcohol consumption and depression or anxiety symptoms in the postpartum period. Such lack of association between poor mental health and risk behaviors in new mothers could be interpreted as resulting from long-term successful policy work and high participation in maternity care in Sweden. This research has clinical relevance as showing that alcohol consumption might not be an indicator of increased risk for PPD in socioeconomically advantaged groups, thereby focusing identification efforts upon other relevant areas. However, to better understand women’s alcohol consumption in the peripartum period, in association with depression and anxiety, future studies need to address these issues in diverse cultural and social contexts.
